# Homes for the Dying

**Published:** 1896-07-18

**Authors:** 


### HOMES FOR THE DYING.

### I.—GENERAL CONSIDERATIONS.

Nearly thirty years ago, when superintendent of a large hospital in the provinces, the writer was asked to attend a meeting of the medical staff. The business was to consider a proposal by one of the physicians, the late Dr. Mackay, which had been favourably entertained by the committee of management, to set apart one ward to which patients could be removed immediately before death. An interesting discussion took place, the objections to deaths in a large ward being forcibly put from the point of view of the other patients and of the one whose end was near. It was felt there was much to be said in favour of the proposal, and ultimately it was agreed to ask the writer to make arrangements for the preparation of a special ward, to be known as the Ward for the Dying. We took no part in the discussion, but, being asked to take the necessary steps with the object in view, we ventured to raise this question—Is each member of the medical staff prepared to certify which of his patients are to be removed to the Ward for the Dying, and if so, are they prepared to face the consequences of a removal of a patient to such a ward who may ultimately recover? This question brought matters to a climax, and from that day to this no further steps have been taken to open such a ward as was then proposed.

Speaking to one of the most active hospital chairmen in London the other day, we happened to mention that we had recently inspected the two existing homes for the dying, Friedenheim and the Clapham Home. He immediately said, "I do not believe in these institutions, because I believe that the cases they provide for can be just as well treated in the poor law infirmaries." We pointed out that his institution sent a number of cases each year to Friedenheim, which cut away the grounds of his argument. "Not at all," was his reply. "Friedenheim exists, is willing to take these cases, and the patients' friends desire that they should go there, but in my view it would be better to send them to the poor law infirmary." On the other hand, Miss F. M. Davidson, the honorary superintendent of Friedenheim, states that her experience is that such a home is not only desirable but necessary, and that it proves of the greatest benefit morally, spiritually, and on every ground of humanity to the patients who enter into it and to their friends. It cannot, however, be denied that the tendency is to admit a number of patients to this class of institution who cannot be said to be in a dying state at all, and it happens in a certain number of instances that the patients admitted so far recover that they are ultimately sent out as convalescent.

To solve this aspect of the problem we took our way to the Free Home for the Dying, 82, The Chase, Clapham, where the sister-in-charge, one of the nursing sisters from St. Margaret's, East Grinstead, helped us to a solution. She gave an account of the trouble she had recently had with an Irishwoman who was sent in to the home from one of the hospitals, suffering from a mortal ailment. This woman's place of residence was in the East of London, and she, poor thing, was such an habitual grumbler, and her habits were of such a character, that it was found impossible to retain her at Clapham. As the result of several years' experience, the sister-in-charge said that she was convinced that if homes for the dying were ever to be of real practical use in the social economy of this country the patients must be selected from the classes above those who properly find their way to the poor law infirmaries, because there was more real suffering and misery amongst governesses, school teachers, the poorer members of the professional class, and other persons in a similar position, than in the classes immediately below them, for which latter the poor law, on the whole, made the best possible provision.

A Hostel of Peace.

The subject of this article has been in our minds for many years. Much thought, careful inquiry, and a full consideration of all the facts have brought us to the conclusion that there is room for a Hostel of Peace, provided it is established upon right principles, and conducted under regulations which will secure that its benefits shall be strictly confined to those who really need such a retreat when their work in this world is over and the end of their life is in sight. Such a home should be confined entirely to cases which cannot secure a suitable and quiet resting-place elsewhere. We have sufficiently indicated the classes who specially need such a Hostel of Peace. They, poor things, have usually little or no means, and are reduced to the direst stress through poverty for which they cannot properly be held responsible. To them such a hostel, coming, as it would do, after years of struggle and stress, must prove, indeed, a very haven of refuge, a valley of happiness, and a ladder of hope. Here, no doubt, there is then a field which those who have money to bestow upon objects of approved utility might profitably cultivate to the blessing of a large number of very worthy, but very poor, people. The establishment of a Hostel of Peace, such as that we have ventured to describe, must produce showers of blessings upon its founders, for, if rightly conducted, it should teach the great truth that death, after all, in the happiest circumstances, is to be rejoiced over, and possibly even to be welcomed by the man or woman whose work is done. We have always thought and felt that, instead of the grim accompaniments which characterise funerals, if the people who follow their beloved to their last resting-places really and truly believed in the religious principles which they profess, then a funeral would be, as it ought to be in most cases, an occasion where brightness, rather than gloom, should be the prevailing note. The grief of those who are left behind, however natural, if really analysed to its depths, must be held to originate in pity for oneself as a survivor rather than in sorrow for the departed who has passed away in the blessed hope of a joyful resurrection. Hence the Hostel of Peace, as a haven of Godlike quietude and reflection, must become in practice so blessed to its inmates that the whole place, if rightly conducted, will ever assume an atmosphere of most cheerful happiness and assured comfort.

(*To be continued.*)

